# The relationship among NICU stressors and irritable bowel syndrome in parents during their infant stay: the mediating role of anxiety

**DOI:** 10.1186/s13052-025-02121-5

**Published:** 2025-09-29

**Authors:** Carmine Vincenzo Lambiase, Michela Guiso, Marcella Pesce, Maria Vendemmia, Letizia Capasso, Giovanni Sarnelli, Francesco Raimondi

**Affiliations:** 1 Department of Humanities, University of Naples Federico II, Via Porta di Massa 1, Naples, 80133 Italy; 2 Division of Neonatology, Department of Translational Medical Sciences, University of Naples Federico II, Via Sergio Pansini 5, Naples, 80131 Italy; 3 Unit of Digestive and Nutritional Pathophysiology, Department of Clinical Medicine and Surgery, University of Naples Federico II, Via Sergio Pansini 5, Naples, 80131 Italy

**Keywords:** Gut-brain interaction, Irritable bowel syndrome, Parents, Mothers, Fathers, NICU, Infants, Stress, Psychosomatic distress, Anxiety

## Abstract

**Background:**

An infant requiring admission to the Neonatal Intensive Care Unit (NICU) is frequently associated with parental stress and anxiety. Irritable bowel syndrome (IBS) represents one of the most common disorders of gut-brain interaction in adult population. The primary objective of this study was to evaluate IBS symptoms in mothers and fathers of NICU infants during hospitalization. Secondary objective was to explore the relationship among NICU stressors, anxiety and IBS symptoms.

**Methods:**

Cross-sectional study. Eighty parents (mothers = 44, fathers = 36) of NICU hospitalized infants filled out validated questionnaires on IBS symptoms, anxiety and NICU stressors (i.e., Sights and Sounds, Infant’s Look and Behaviour, Parental Role Alterations). Student’s t test and Chi-square test were used to compare maternal and paternal IBS symptoms, prevalence and severity. The mediating effect of anxiety among NICU stressors and IBS was tested using mediation analysis.

**Results:**

Mothers scored above the clinical cut-off for IBS more frequently than fathers (59.1% vs. 44.6%). Symptoms were significantly higher in mothers as compared to fathers (*p* = .021). Anxiety fully mediated the effect of Sights and Sounds (β = 0.147, SE = 0.058, 95% CI: [ 0.046, 0.277]), Infant Look and Behaviour (β = 0.117, SE = 0.049, 95% CI: [0.024, 0.215]) and Parental Role Alterations (*β* = 0.132, SE = 0.050, 95% CI: [0.044, 0.241]) on IBS symptoms.

**Conclusions:**

Our study demonstrated for the first time that parents of NICU infants experience IBS symptoms during hospitalization and distinguished the somatic experience among mothers and fathers during their infant NICU stay. Parental experience of NICU hospitalization deserve to be studied as a potential stressful life event implying both psychological and somatic distress. Integrating tailored stress-reduction interventions sensitive to gender differences into Family Centered-Care practices is essential to reduce parental distress and support parental involvement during NICU hospitalization.

## Introduction

Stress is a process between an individual and a stressor [1]. Individuals who appraise stressors as overwhelming their resources will experience distress [2]. The preterm distress model was specifically developed within the context of the Neonatal Intensive Care Unit (NICU) [3]. This model detected the following as the most prominent stressors: Sights and Sounds, Infant’s look and behaviour and Parental role alterations [3, 4]. The preterm distress model guided the vast majority of the research on the experienced psychological distress of parents during NICU hospitalization [5, 6]. However, little attention was given to the somatic distress of parents during NICU hospitalization and the interplay among psychological and somatic distress.

Irritable bowel syndrome (IBS) is one of the most common disorder of gut-brain interaction (DGBI) ranging from 5-to-10% within the general population [7]. IBS is 1.5 times more common in women as compared to men [8]. It is a symptom-based condition defined by the presence of abdominal pain or discomfort, with altered bowel habits [9]. Recently, guidelines emphasized the understanding of IBS etiology and treatment within a biopsychosocial perspective [10, 11].

The biopsychosocial model underlines the interplay among biological and psychological factors in determining well-being [12, 13]. Several studies demonstrated that psychological distress influence IBS symptoms [14, 15]. Anxiety is one of the most determinant components of psychological distress leading to IBS onset [16]. Therefore, adopting a biopsychosocial perspective including the context-related NICU stressors, anxiety and IBS symptoms is important to expand knowledge on psychosomatic distress response in parents of NICU hospitalized infants [17, 18]. To the best of our knowledge, no studies investigated IBS prevalence and severity in mothers and fathers of NICU hospitalized infants. Therefore, the main objective of this study is to evaluate IBS symptoms in mothers and fathers of NICU infants during hospitalization.

According to the above-mentioned scientific literature on IBS and gender differences the following hypothesis was proposed:

### Hypothesis 1 (H1)

Mothers will experience higher levels of IBS symptoms than fathers. 

Based on the above-mentioned literature investigating factors that influence IBS symptoms we stated the following hypothesis:

### Hypothesis 2 (H2)

*NICU Stressors and Anxiety will exacerbate IBS symptoms in parents of NICU infants.*

### Hypothesis 3 (H3)

**Fig. 1:**
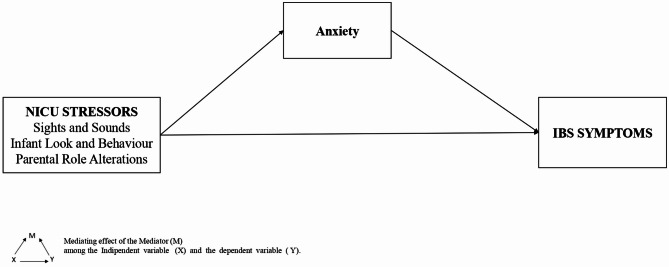
The hypothesized mediation model

*Anxiety will mediate the effect among NICU stressors and IBS symptoms (*Fig. 1*).*

### Methods

The Revised Standards for Quality Improvement Reporting Excellence (SQUIRE 2.0) guidelines were used to report this cross-sectional, single-centre study. The study was approved by the local Ethics Committee (protocol number: 126/24) and parents filled in written informed consent to participate to the study. The study was in accordance with the provisions of the 1995 Declaration of Helsinki and subsequent modifications. Parents were informed about the scope of the study by a research psychologist and asked to fill out standardized questionnaires from February to June 2024.

Sociodemographic information about parents (i.e., parental age, education level, being first time parent and months from NICU admission to questionnaire administration) and infant characteristics (i.e., gestational age, birthweight. being twin) were collected.

The Parental Stressor Scale: Neonatal Intensive Care Unit (PSS: NICU; [19]) encompasses 34 items divided into 3 subscales: Sights & Sounds (α = 0.83; ω = 0.83), Infant Look and Behaviour (α = 0.93; ω = 0.93) and Parental Role Alterations (α = 0.86; ω = 0.86) and a general stress item. Parents had to rate each item on a 5-point Likert scale ranging from 1 (not at all stressful) to 5 (extremely stressful) according to their stress at time of administration. An overall stress score is computed from the three subscales ranging from 0 to 5. PSS: NICU scores can be computed according to Miles et al. [19], using two methods: (a) the Stress Occurrence Level (SOL) calculated including only experienced items and (b) the Overall Stress Level (OSL) scoring “not applicable items” with one point. As our focus of interest was related to parental real lived experience, we used the SOL method to capture the relationships among NICU stressors and the other study variables. For this study the SOL score had excellent internal reliability (α = 0.95; ω = 0.95).

Self-rating anxiety scale (SAS [20]; α = 0.85; ω = 0.82) is 20-item-self-report questionnaire that evaluate anxious symptoms. The items are related to the week before questionnaire administration, with higher scores corresponding to higher levels of anxiety. Scores < 50 indicate absence of psychopathological disturbances, whilst anxiety based on the results of the questionnaires are labelled as mild (score ranging from 50–to-59), moderate (score ranging from 60–to-69) and severe (score ≥ 70).

IBS-Symptom Severity (IBS-SSS; [21]) evaluates presence and severity of gastrointestinal symptoms. The IBS-SSS (α = 0.75; ω = 0.85) ranges from 0 to 500 with higher scores indicating more severe symptoms. Subjects can be categorized as having none (< 75), mild (75–175), moderate (176–300), or severe (> 300) IBS symptoms.

### Data analysis

Data were analyzed with Statistical Package for the Social Sciences (SPSS v.29 software [22]). Data normal distribution was established evaluating Asymmetry and Kurtosis, values ranging from − 1.5 to + 1.5 were considered for normally distributed variables [23]. Two-tails correlations were computed using Pearson’s coefficient *r* to test relationships among sociodemographic variables, infant characteristics, NICU stressor, anxiety and IBS. Mean differences and prevalence between groups were computed respectively using t-test and Chi-square test. Cohen’s *d* and Cramer’s V were used to measure effect sizes. PROCESS macro 4.2 for SPSS [24] was used to test mediation models among NICU stressors, anxiety and IBS. The statistical significance of the total indirect effect of mediating variables was examined using bootstrapping methods to estimate bias-corrected asymmetric confidence intervals (CIs) with 5000 resamples with replacement. In each mediation model, one NICU stressor (i.e., Sights and Sounds, Infant’s Look and Behaviour and Parental Role Alterations) was alternatively chosen as the independent variable (X), Anxiety (M) was chosen as mediator while IBS was used as dependent variable (Y).

## Results

Eighty parents (mothers *N* = 44; fathers *N* = 36) of 51 infants participated to the study. Descriptive data of the included parents are reported in Table 1 while infant characteristics (*N* = 51) are displayed in Table 2.

**Table 1:** Comparisons among parental sociodemographic information

	Mothers (*N* = 44)	Fathers (*N* = 36)	*p*
**Age**			
Mean (SD) Median Range	33.8 (6) 32 27;49	38.5 (8.6) 36 27;60	0.19
**Educational level**			
Middle school High school University Missing	4 (9.1%) 19 (43.2%) 8 (18.2%) 13 (29.5%)	4 (11.1%) 14 (38.9%) 7 (19.4%) 11 (30.6%)	0.91
**Administration time (days)**			
Mean (SD) Median Range	23.5 (24.5) 14 2;111	24.4 (26.2) 14 2;111	0.87

*Student’s t* test was used for mean differences. Chi-square test was used for frequencies

**Table 2:** Neonatal characteristics of the enrolled infants (*N* = 51)

	*N*(%)	Mean (SD)	Median	range
**Sex**				
Female	16(31.3)			
**Gestational age (GA)**		31.4 (4.1)	32	22; 40
Full term Late preterm Moderate preterm Very preterm Extremely preterm	4(8.8) 8(17.7) 12(26.6) 15(33.3) 9(20)			
**Birthweight**		1525.8 (573)	1440	480; 3190
Normal birthweight LBW VLBW ELBW	5(11.1) 14(31.1) 15(33.3) 11(24.4)			
**Twins**	6(13.3)			

Preliminary analyses showed that the three NICU stressors correlated with anxiety (Sights and Sounds *r* = .483 *p* < .001; Infant Look and Behaviour *r* = .453 *p* < .001; Parental Role Alterations *r* = .443 *p* < .001) and IBS symptoms (Sights and Sounds *r* = .277 *p* = .014; Infant Look and Behaviour *r* = .339 *p* = .002; Parental Role Alterations *r* = .278 *p* = .014). Anxiety positively correlated with IBS symptoms (*r* = .372 *p* < .001). No sociodemographic data or infant characteristics correlated with IBS (*p* > .05).

### H1

Mothers scored above the clinical cut-off for IBS more frequently than fathers (59.1% vs. 44.6%). Mean IBS symptom scores were significantly higher in mothers as compared to fathers (*t*(78) = 2.364, Cohen’s D = 0.515, 95% CI[0.081;0.978], *p* = .021). IBS-SSS item comparison (Table 3) revealed that mothers experienced significantly higher presence of abdominal pain (Cramer’s V = 0.306, χ^2^ = 7.480, *p* = .006) and distension (Cramer’s V = 0.247, χ^2^ = 4.869, *p* = .027) than fathers.

**Table 3:** Comparison of IBS score, severity, incidence and IBS-SSS items among mothers and fathers

	Mothers (*N* = 44)	Fathers (*N* = 36)	*p*
IBS score Mean (SD)	114 (90)	72.6 (59.6)	0.02**
IBS ≥ 75	26 (59.1%)	16 (44.4%)	0.19
IBS severity Mild Moderate Severe	16 (36.3%) 8 (18.8%) 2 (4.5%)	13 (36.1%) 3 (5.5%) 0 (0%)	
Presence of abdominal pain N (%)	13 (29.5%)	2 (5.5%)	0.00***
Severity of abdominal pain Mean (SD)	49.2 (18.8)	40(14.1)	0.51
Number of days suffering of abdominal pain out of 10 day-time period* Mean (SD)	3.3 (2.4)	3.6 (3)	0.07
Presence of abdominal distension N (%)	14 (31.8%)	4 (11.1%)	0.03**
Severity of abdominal distension Mean (SD)	49.8 (24)	41 (18.8)	0.42
Bowel habits satisfaction Mean (SD)	43.5 (29.8)	40.6 (30.6)	0.67
IBS impact on life functioning Mean (SD)	32.4 (25.8)	23.4(20.6)	0.08

*Data reported refer to subjects who answered ≥ 1 day

** *p* < .05

*** *p* < .01

### H2

Sights and Sounds (β = 0.483, 95% CI: [3.204,7.841], *p* = .00), Infants Look and Behaviour (β = 0.453, 95% CI: [2.477,6.617], *p* = .00) and Parental Role Alterations (β = 0.443, 95% CI: [3.212,8,875], *p* = .00) predicted Anxiety. Moreover, Sights and Sounds (*R*^*2*^ = 0.077, β = 0.277, 95% CI: [4.542,38.986], *p* = .01), Infants Look and Behaviour (*R*^*2*^ = 0.115, β = 0.339, 95% CI: [8.653,38.652], *p* = .00) and Parental Role Alterations (*R*^*2*^ = 0.080, β = 0.278, 95% CI: [5.525,46.612], *p* = .01) predicted IBS symptoms as well. Finally, Anxiety predicted IBS symptoms (*R*^*2*^ = 0.138, β = 0.372, 95% CI: [1.082,3.961], *p* < .00).

### H3

**Fig. 2:**
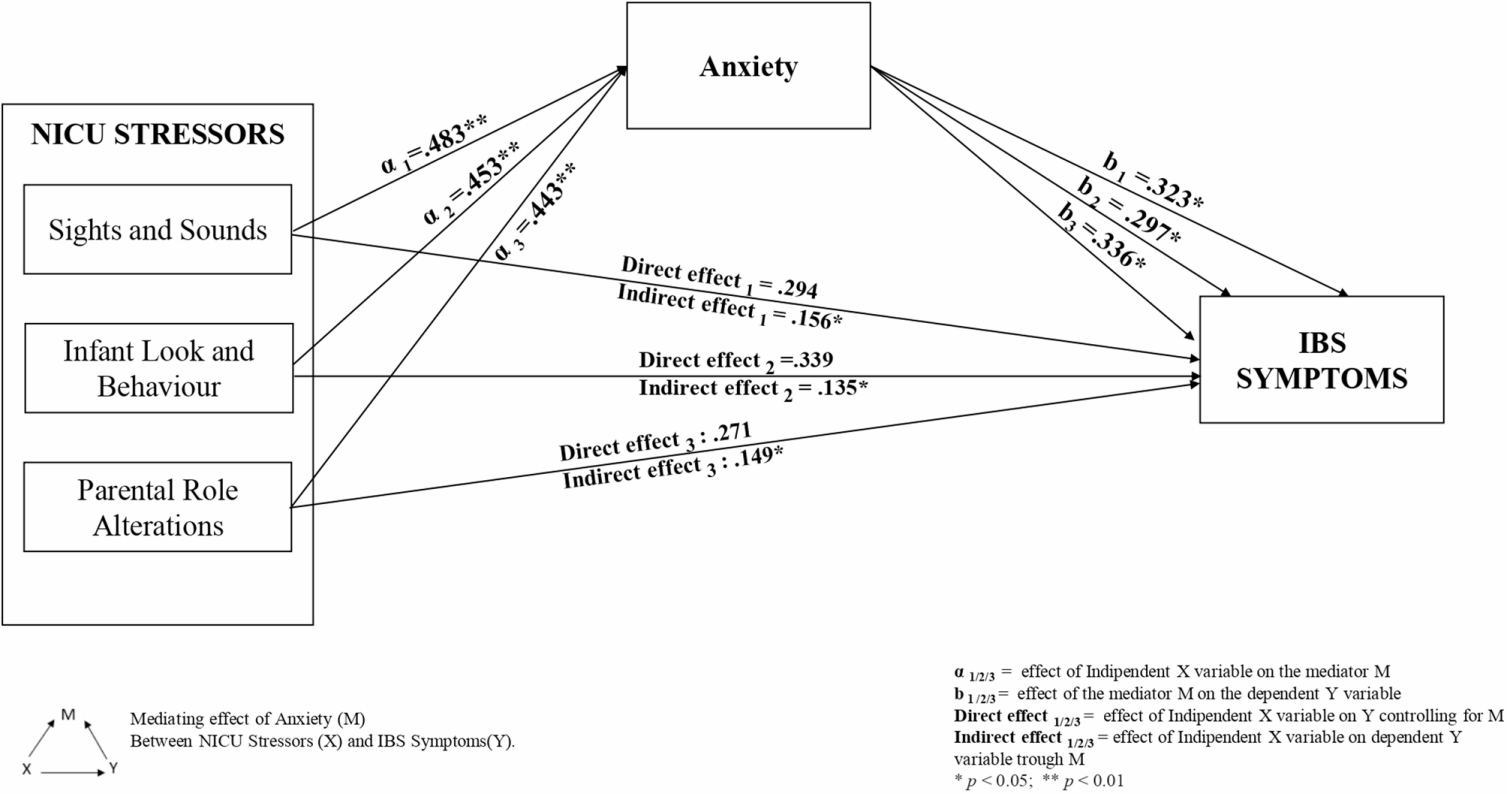
The summary of the mediation models among NICU stressors, anxiety and IBS symptoms in parents of NICU hospitalized infants

As the assumptions for the mediation model were confirmed (i.e., X→Y; X→M; M→Y) we could proceed to test the mediating effect of Anxiety among each NICU stressor (i.e., Sights and Sounds, Infant Look and Behaviour and Parental Role Alterations) and IBS symptoms. Confirming our hypothesis, anxiety fully mediated the effect of Sights and Sounds (β = 0.147, SE = 0.058, 95% CI: [ 0.046, 0.277]), Infant Look and Behaviour (β = 0.117, SE = 0.049, 95% CI: [0.024, 0.215]) and Parental Role Alterations (= 0.132, SE = 0.050, 95% CI: [0.044, 0.241]) on IBS symptoms. The summary of the three mediation models is displayed in Fig. 2.

## Discussion

This study primary aimed to examine the prevalence, severity and characteristics of IBS in parents according to a biopsychosocial perspective sensitive to gender differences. To the best of our knowledge, our study demonstrated for the first time that NICU hospitalization can be a precipitating factor to IBS onset in parents of NICU infants.

In particular, maternal IBS symptoms were higher than paternal ones and above the clinical cut-off. Our findings are in line with the epidemiology of IBS that is higher in females than males [25]. Importantly, somatic distress related to IBS may reflect the psychological distress that is usually higher in mothers as compared to fathers [26, 27].

Among the explaining factors, gender norms could play a key role to IBS development [28]. In particular the lack to achieve culturally accepted gendered norms as mothers (e.g., taking care of their infant better than anyone else) [29] would contrast with the maternal feelings of exclusion from infant care experienced during NICU hospitalization [30]. This discrepancy may exacerbate maternal IBS symptoms during NICU hospitalization and activate a vicious circle of psychosomatic distress [31] with cascading effects on family and work dynamics [32].

Secondly, our study specifically investigated the relationship among NICU stressors, anxiety and IBS. Importantly, we found that anxiety fully mediated the effect of the three NICU stressors on IBS confirming that anxiety is a risk factor for IBS symptoms [33]. Our findings enrich the comprehension of the interactions among NICU stressors and psychological distress leading to IBS development in parents of NICU infants. Targeting parental anxious symptoms due to NICU hospitalization using multi-layered interventions including Family-Centred Care practices, stress reduction strategies and cognitive-behavioural therapy may also stop the vicious circle of IBS symptom perpetuation [34, 35].

Our study contributes to distinguish somatic experience among mothers and fathers during their infant NICU hospitalization. Psychoeducation and counselling on the psychological, somatic and social consequences following admission (e.g., NICU environment, developmental care and parental role expectations rather than actual possibilities) are warranting to inform and enable mothers and fathers to cope with the variety of reactions following NICU hospitalization. Moreover, education of healthcare providers is essential to enhance their understanding of the subtle gender norms that mothers and fathers have to face to during NICU hospitalization in order to boost the effectiveness of Family-Centered Care practices. Finally, policy makers and working managers should be aware of the potential family and work decrements experienced by parents during NICU hospitalization in order to avoid their financial distress [36].

Among the strengths of this study, we argue the description of IBS symptoms in both mothers and fathers of NICU hospitalized infants according to a biopsychosocial perspective sensitive to gender differences. Further, our study was able to capture the relationship among NICU stressors, anxiety and IBS improving mechanisms understanding and providing the opportunity to implement tailored interventions [11]. Among the limitations of our study, we acknowledge the relatively small sample size. Future studies could investigate factors mitigating IBS symptoms in mothers and fathers during NICU hospitalization.

## Conclusions

For the first time this study explored IBS in parents of NICU hospitalized infants. Importantly, this study newly emphasizes a specific pattern for IBS development in parents during NICU hospitalization. Further, our findings elucidated the somatic distressful experience of mothers and fathers during NICU stay of their own infant and its interplay with psychological distress. This study also provides valuable suggestions to improve parent-staff interactions. NICU hospitalization deserves to be studied as a stressful life experience implying psychological and somatic distress in parents and changes in family and work dynamics within a biopsychosocial model. Implications of these findings include the need to provide tailored stress-reduction interventions sensitive to gender differences in order to reduce parental distress and support parental involvement during NICU hospitalization.

## Data Availability

The datasets used and/or analysed during the current study are available from the corresponding author on reasonable request.

## Abbreviations

ELBW: Extremely low birthweight
GA: Gestational age
GBID: Gut-brain interaction disorders
LBW: Low birthweight
IBS: Irritable bowel syndrome
IBS-SSS: IBS-symptom severity
NICU: Neonatal intensive care unit
OSL: Overall stress level
PSS:NICU: Parental stressor scale: neonatal intensive care unit
SAS: Self-rating anxiety scale
SOL: Stress occurrence level
SPSS: Statistical package for the social sciences
VLBW: Very low birthweight
